# Magnetic Marking and Intraoperative Detection of Primary Draining Lymph Nodes in High-Risk Prostate Cancer Using Superparamagnetic Iron Oxide Nanoparticles: Additional Diagnostic Value

**DOI:** 10.3390/molecules22122192

**Published:** 2017-12-09

**Authors:** Alexander Winter, Svenja Engels, Lena Reinhardt, Clara Wasylow, Holger Gerullis, Friedhelm Wawroschek

**Affiliations:** University Hospital for Urology, Klinikum Oldenburg, School of Medicine and Health Sciences, Carl von Ossietzky University Oldenburg, D-26111 Oldenburg , Germany; engels.svenja@klinikum-oldenburg.de (S.E.); lena@buecken-reinhardt.de (L.R.); claraw98@gmx.de (C.W.); gerullis.holger@klinikum-oldenburg.de (H.G.); wawroschek.friedhelm@klinikum-oldenburg.de (F.W.)

**Keywords:** superparamagnetic iron oxide nanoparticles (SPION), prostate cancer, sentinel node, magnetometer, lymphadenectomy

## Abstract

Sentinel lymph node dissection (sLND) using a magnetometer and superparamagnetic iron oxide nanoparticles (SPIONs) as a tracer was successfully applied in prostate cancer (PCa). Radioisotope-guided sLND combined with extended pelvic LND (ePLND) achieved better node removal, increasing the number of affected nodes or the detection of sentinel lymph nodes outside the established ePLND template. We determined the diagnostic value of additional magnetometer-guided sLND after intraprostatic SPION-injection in high-risk PCa. This retrospective study included 104 high-risk PCa patients (PSA >20 ng/mL and/or Gleason score ≥ 8 and/or cT2c) from a prospective cohort who underwent radical prostatectomy with magnetometer-guided sLND and ePLND. The diagnostic accuracy of sLND was assessed using ePLND as a reference standard. Lymph node metastases were found in 61 of 104 patients (58.7%). sLND had a 100% diagnostic rate, 96.6% sensitivity, 95.6% specificity, 96.6% positive predictive value, 95.6% negative predictive value, 3.4% false negative rate, and 4.4% false positive rate (detecting lymph node metastases outside the ePLND template). These findings demonstrate the high sensitivity and additional diagnostic value of magnetometer-guided sLND, exceeding that of ePLND through the individualized extension of PLND or the detection of sentinel lymph nodes/lymph node metastases outside the established node template in high-risk PCa.

## 1. Introduction

Lymph node (LN) status is a crucial and therapeutically relevant prognostic factor for prostate cancer (PCa). Using LN status, the risks of progression can be calculated and appropriate adjuvant therapy can be planned. There is increasing evidence for the positive therapeutic effects of pelvic LN dissection (PLND) or resecting LN metastases, particularly in patients with minimal LN invasion (LNI) [1,2,3,4]. Despite recent advances in imaging, PLND remains the most reliable method for LN staging in clinically localized PCa. However, the reliability of these procedures is limited by their spatial resolution, which limits the sensitivity (49–66%) of detecting LN (micro)metastases [5].

The prevalence of LNI is directly associated with the number of dissected LNs or the extent of PLND [6]. Therefore, the European Association of Urology guidelines recommend an extended PLND (ePLND) approach for LN staging in PCa patients with a >5% risk of LNI [7].

Because of the increased complication rate of ePLND and the low detection rate of limited PLND, Wawroschek et al. transferred techniques and concepts of targeted radioisotope-guided sentinel LN (SLN) identification in other tumor entities to PCa [8,9,10]. In a recent systematic literature review, the diagnostic accuracy of sentinel-guided LN dissection (sLND) was determined by evaluating data from 21 studies (2509 patients). These findings revealed that the diagnostic accuracy of targeted sLND and ePLND were almost the same. sLND combined with ePLND achieved better node removal by increasing the number of affected nodes in 5% of cases, indicating therapeutic potential [11]. Accordingly, it was demonstrated that sLND yielded higher LNI rates in sentinel cohorts than was expected from established nomograms [12,13,14]. One reason for this might be the advantage of targeted dissection of tumor-associated LNs or tailoring the extent of PLND to individual lymphatic drainage. Joniau et al. showed that sLND could have an additional diagnostic value over and above ePLND [15]. They showed that 8% of LN-positive patients would have been missed if only a standard ePLND had been performed.

Nevertheless, due to the ionizing radiation emitted by the technetium-based tracer material, the advantages of the current SLN procedure are accompanied by serious downsides. The dependence on radioisotopes limits the application of this procedure to small parts of the developed world and imposes restrictions on patient planning and hospital logistics. Moreover, the procedure exposes patients and surgical staff to radiation. To overcome these impediments, superparamagnetic iron oxide nanoparticles (SPIONs) have been successfully applied to identify SLNs in breast cancer patients [16,17]. Recently, we presented the first results of intraoperatively detecting SLNs in PCa using a system that comprises a magnetic tracer and a handheld magnetometer [18].

In view of these findings, we hypothesized that magnetometer-guided sLND could have additional diagnostic value because it allows the targeted removal of affected LNs, especially outside the established PLND template in high-risk PCa patients.

To assess the diagnostic accuracy of magnetometer-guided sLND in high-risk PCa, we analyzed high-risk PCa patients from a prospective cohort who underwent radical prostatectomy with magnetometer-guided sLND after intraprostatic SPION injection and ePLND. The diagnostic accuracy of magnetometer-guided sLND was determined using ePLND as a reference standard.

## 2. Results

This study included 104 high-risk PCa patients who underwent radical retropubic prostatectomy with ePLND and magnetometer-guided sLND after intraprostatic injection of SPIONs. Table 1 summarizes the patient characteristics. Median total PSA was 17.21 ng/mL (interquartile range (IQR) 8.23–32.56).

**Figure 1:**
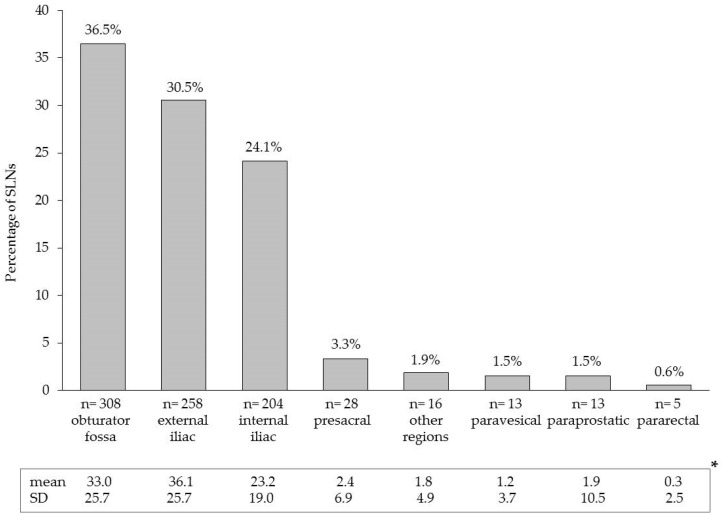
Anatomical distribution of the 845 prostate sentinel lymph nodes from the 104 high-risk patients based on intraoperative magnetometer-guided detection after intraprostatic injection of superparamagnetic iron oxide nanoparticles. * Mean values and standard deviations (SD) on patient level.

SLNs were successfully detected by magnetometer-guided sLND in all patients (104/104), resulting in a diagnostic rate of 100%. A total of 845 SLNs were identified. The median number of detected SLNs was 8 (IQR 5–11). SLNs were also localized outside the established ePLND template (e.g., presacral region: 3.3% and paravesical region: 1.5%). Figure 1 shows the detailed distribution of all SLNs per anatomic region.

Taking the ePLND template as a reference standard, sLND results had a 100% diagnostic rate, 96.6% sensitivity, 95.6% specificity, 96.6% positive predictive value (PPV), 95.6% negative predictive value (NPV), and a 3.4% false negative rate.

**Figure 2:**
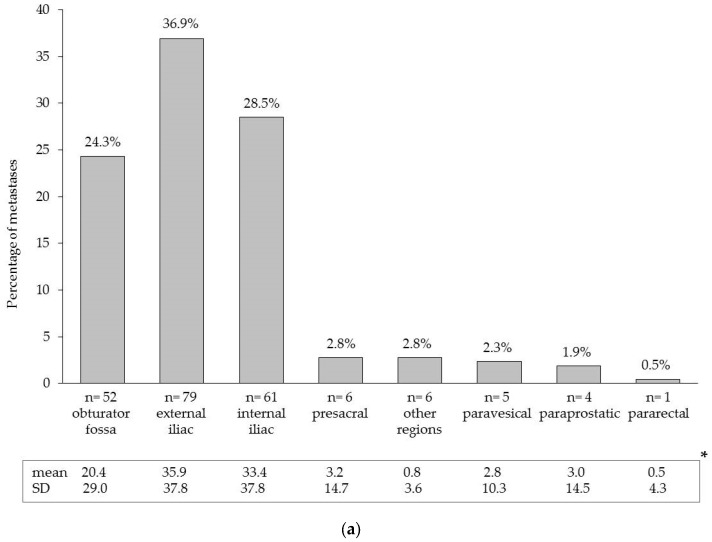

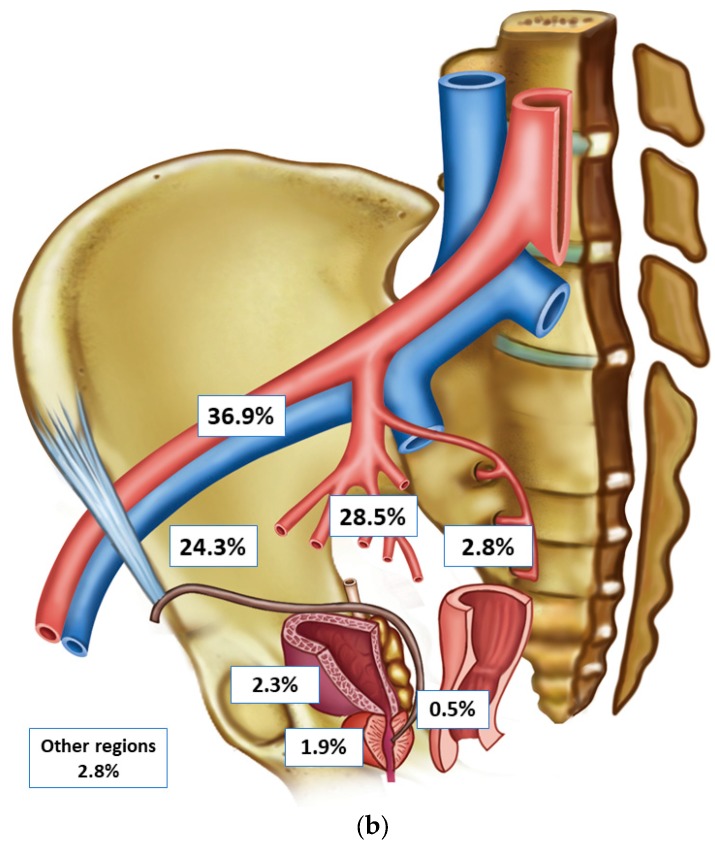
(**a**) areas and anatomical distribution of lymph node metastases (*n* = 214) detected by extended pelvic lymph node dissection and/or magnetometer-guided sentinel lymphadenectomy after intraprostatic injection of superparamagnetic iron oxide nanoparticles in 61 lymph node-positive patients with high-risk prostate cancer; (**b**) distribution and localization of lymph node metastases in an anatomical pelvic model. * Mean values and standard deviations (SD) on a patient level.

LN metastases were found in 61 of 104 patients (58.7%), with a median of two positive LNs (IQR 1–4). Figure 2 shows the distribution of all detected LN metastases per anatomic region.

sLND showed an additional diagnostic value in two of the 61 LN-positive patients. In these two cases, sLND detected two LN metastases outside the ePLND template, while ePLND did not reveal any metastases (false positive rate 4.4%). The percentage of LN-positive patients with only SLN metastases was 55.7% (*n* = 34).

## 3. Discussion

The intraoperative use of a handheld magnetometer in combination with SPION-based magnetic resonance imaging after intraprostatic SPION-injection provides a new, entirely radiation-free technique for identifying SLNs in PCa [18,19]. Using this magnetic-targeted sentinel approach, we showed an additional diagnostic value over and above ePLND in high-risk PCa patients. Without the individualized extension of PLND using magnetic sLND, two LN-positive patients (3.3% of all LN-positive patients) would not have been identified.

In this study, which included only high-risk PCa patients, SLNs were intraoperatively identified in all patients. In pilot studies including patients with intermediate-risk and high-risk PCa, we successfully identified SLNs in 89.5% and 100% of cases, respectively, using the same magnetic technique [18,20]. For radioisotope-guided sLND, Holl et al. showed a detection rate of 98.0% in a study that included over 2000 low-risk, intermediate-risk, and high-risk PCa patients [21]. A meta-analysis revealed a pooled detection rate of 93.8% for radio-guided sLND [22]. In a current systematic literature review, the median cumulative percentage for the detection rate was 95.9% (IQR 89.4–98.5%) [11]. Accordingly, the magnetic sLND technique works even in high-risk PCa.

However, one fundamental problem of the SLN approach is that when LNs are fully metastasized or when the lymph pathways are blocked, the afferent lymph will be redirected to other LNs/non-SLNs [23]. These LNs cannot be identified using the sentinel procedure, resulting in false-negative findings. The false-negative rate was shown to correlate with Gleason scores [21]; patients with high-risk disease could thus have both positive SLNs and positive non-SLNs or LNI only in non-SLNs. In a study by Weckermann et al., only positive non-SLNs were identified (false-negative results) by radioisotope-guided sPLND in two of 96 men with positive LNs (2.1%) [24]. Therefore, a lower median sensitivity and a higher false-negative rate was observed in studies including only patients at higher risk of LNI [11]. In this study, two of 61 LN-positive patients were not detected by magnetometer-guided sLND, and 44.3% of LN-positive cases had SLN metastases and additional metastases in non-SLNs.

Conversely, this and other studies have shown an increased detection rate of positive LNs when combining ePLND with sLND or the individualized extension of LN dissection outside the borders of ePLND. For example, Joniau et al. showed that 21% of preoperatively detected SLNs could be found in the presacral and pararectal region. Moreover, 8% of LN-positive patients would have been missed if an LN dissection in the presacral region had not been performed [15]. Accordingly, a significant number of SLNs could be visualized outside the standard node template in studies dealing with the radioactive marking approach [25,26]. Through the use of magnetic marking, a high proportion of SLNs could be visualized outside the established ePLND template. In total, 24% of SPION-marked nodes were found, one half each in the presacral and pararectal regions [19]. Results of a current systematic review indicate that one in 20 patients who undergo ePLND, metastatic LNs would have been left behind without performing sLND [11].

As shown in a recent systematic review, there is increasing evidence that surgically removing lymphatic metastases may lead to more favorable outcomes [9]. Thus, there are good arguments for combining sLND with ePLND in high-risk PCa. However, further studies, including a long-term follow-up study, are required to explore the effects of (additional) magnetometer-guided sLND and other SLN techniques on oncologic outcomes.

## 4. Materials and Methods

### 4.1. Patients

This study included 104 consecutive patients with high-risk PCa (European Association of Urology risk group: PSA >20 ng/mL and/or Gleason Score ≥ 8 and/or cT2c [27]) who underwent radical retropubic prostatectomy with magnetometer-guided sLND after intraprostatic injection of SPION and ePLND, which were performed by two highly experienced surgeons at our university center between February 2015 and September 2017. Patients were identified in a prospectively accrued study population. In total, this cohort included 182 patients with intermediate- and high-risk PCa, who had received such intervention. After exclusion of intermediate-risk patients, 104 patients remained. Written informed consent was obtained from all patients.

### 4.2. Magnetic SPION Tracer

The SPION tracer (Sienna+^®^) used in these studies is a component of the SentiMag^®^ system (Endomagnetics Ltd., Cambridge, UK). The system for marking and identifying SLNs comprises a handheld magnetometer, the SentiMag^®^ unit, and the Sienna+^®^ magnet tracer. Both are CE certified as class IIa medical devices. The particles have a carboxydextran coating and a mean hydrodynamic diameter of 60 nm. Sienna+^®^ has comparable functional properties to that of ^99m^Technetium nanocolloid because, upon interstitial injection, the tracer flows just like the radio nuclide through the lymph system and gets trapped in SLNs.

### 4.3. Tracer Injection

The sentinel technique in PCa differs from those of other tumor types. In breast cancer and malignant melanoma, a well-directed peritumoral injection is only placed to observe the lymphatic drainage of the tumor. In PCa, which commonly occurs as a multifocal malignancy, it is not known with absolute certainty from which part of the organ the metastatic spread originated or which is the index lesion. Therefore, the aim of prostate lymph scintigraphy must be imaging all primary draining LNs of the prostate, under which the SLN of cancer also exists.

In this study, one urologist injected 2 mL of SPION (Sienna+^®^) into the prostate of patients using transrectal ultrasound guidance 24 h before surgery. Based on our examinations and those of others, the tracer was evenly spread as three deposits on both sides of the prostate in all cases as described previously [18].

### 4.4. Magnetometer-Guided sLND, ePLND, and Histopathological Examination

Patients underwent magnetometer (SentiMag^®^)-guided sLND and ePLND, followed by radical retropubic prostatectomy. All cases were performed by two high-volume surgeons, who applied the same anatomic template during ePLND. The ePLND template included the area along the external iliac vessels, with the distal limit being the femoral canal. Proximally, ePLND was carried out to and included the bifurcation of the common iliac artery. All lymphatic fatty tissue along the internal iliac artery and within the obturator fossa and the area dorsal of the obturator nerve was removed, as described by Weingärtner et al. [28]. The lateral limit consisted of the pelvic sidewall, and the medial dissection limit was defined by perivesical fat.

During sLND, all metal retractors were removed from the surgical field, and polymer retractors (SUSI^®^, Aesculap^®^; B. Braun Melsungen AG, Melsungen, Germany) were used to avoid interference with the magnetometer when detecting SLNs with the SentiMag^®^ probe. All SLNs detected by the SentiMag^®^ were removed, whereby each magnetically active LN was seen as an SLN. For surgical reasons, LNs other than SLNs directly adjoining and adhering to SLNs were also removed if in situ separation was not possible. In these cases, LNs were macroscopically detected (tactile and visual) ex vivo and separated by the surgeon from each other or from the containing fibro-fatty tissue. Thereafter, ePLND was conducted to remove remaining lymphatic fatty tissue from the above-named regions. Afterwards, LNs were also macroscopically detected and separated by the surgeon from the containing fibro-fatty tissue.

Postoperatively, all LNs were detected and separated by the surgeon (SLNs and non-SLNs), initially cut in 3-mm transverse sections, routinely processed and embedded in paraffin, while 4–5-µm-thick sections were stained with hematoxylin-eosin.

### 4.5. Outcome Measures of Magnetometer-Guided sLND

As established by our and other working groups and in line with the results of a recent international sentinel consensus meeting, diagnostic accuracy of sLND was assessed by using conventional ePLND as a reference standard in the same cohort [11,27,29]. By complying with this standard, the comparability with the results of other sentinel techniques should be achieved.

The outcomes used to analyze diagnostic test accuracy were diagnostic rate (patients with at least one detected SLN/total number operated), sensitivity, specificity, PPV, NPV, false-positive, and false-negative rates; all were measured at the patient level. False-negative cases were defined as patients with histologically-negative SLN, whilst cancer was found in other LNs. False-positive cases were defined as patients with SLNs containing metastases outside the ePLND template, while the ePLND template did not reveal any metastases [11]. Thus, the false-positive rate provides a measure of the additional diagnostic value of sLND over and above ePLND.

A 2 × 2 table with sLND as the index test and ePLND as the reference standard was used to calculate sensitivity, specificity, NPV, and PPV. Additionally, the anatomic distribution of detected LN metastases and identified SLNs were analyzed.

### 4.6. Ethical Approval

All subjects gave their informed consent for inclusion before they participated in the study. The study was conducted in accordance with the Declaration of Helsinki, and the protocol was registered in an international clinical trials register (Research Registry: researchregistry3232). The studies on the prospective cohort were approved by the Ethics Committee of the Medical Chamber of Lower Saxony, Germany (no. 24/2014) and the Medical Ethics Committee of the Carl von Ossietzky University Oldenburg (no. 2017-006).

## 5. Conclusions

Magnetometer-guided sLND after intraprostatic injection of SPIONs was successfully applied in PCa. This work demonstrates the high reliability of this new magnetic sentinel approach in detecting LN-positive patients and an additional diagnostic value exceeding that of ePLND in high-risk PCa. Magnetic sLND combined with ePLND achieves better node removal by increasing the number of affected LNs. Increasing evidence of the therapeutic effects of surgically removing LN metastases speaks to the promise of combining sLND with ePLND in high-risk PCa. Further studies, including ones with long-term follow-up, are required to explore the effects of (additional) magnetometer-guided sLND on patient outcomes.

## Figures and Tables

**Table 1:** Patient characteristics.

	Overall *n* = 104	Patients with Negative LNs *n* = 43 (41.35%)	Patients with Positive LNs *n* = 61 (58.65%)
Age, years (median)	69	69	68
IQR	63–72.5	63.5–72	62–73
Total PSA, ng/mL (median)	17.21	12.77	21.79
IQR	8.32–32.56	7.49–24.27	11–41.27
No. of LNs removed (median)	17	15	17
IQR	12–21	12–20	13–21
No. of SLNs removed (median)	8	9	7
IQR	5–11	6–12	5–11
No. of positive LNs (median)			2
IQR			1–4
Tumor stage (%)			
T1c	23 (22.12)	11 (25.58)	12 (19.67)
T2a	7 (6.73)	3 (6.98)	4 (6.56)
T2b	6 (5.77)	3 (6.98)	3 (4.92)
T2c	49 (47.12)	19 (44.19)	30 (49.18)
T3	19 (18.27)	7 (16.28)	12 (19.67)
Biopsy Gleason score (%)			
6 (3 + 3)	10 (9.62)	8 (18.60)	2 (3.28)
7 (3 + 4)	25 (24.04)	11 (25.58)	14 (22.95)
7 (4 + 3)	20 (19.23)	7 (16.28)	13 (21.31)
≥8	49 (47.12)	17 (39.53)	32 (52.46)
Postoperative Gleason score (%)			
6 (3 + 3)	2 (1.94) *	2 (4.65)	0 **
7 (3 + 4)	23 (22.33) *	20 (46.51)	3 (5.00) **
7 (4 + 3)	35 (33.98) *	11 (25.58)	24 (40.00) **
≥8	43 (41.75) *	10 (23.26)	33 (55.00) **
Pathologic stage (%)			
pT2	28 (26.92)	26 (60.47)	2 (3.28)
pT3a	22 (21.15)	10 (23.26)	12 (19.67)
pT3b	50 (48.08)	7 (16.28)	43 (70.49)
pT4	4 (3.85)	0	4 (6.56)

IQR, Interquartile range; (S)LN, (sentinel) lymph node; PSA, prostate specific antigen; */**, data are based on a population of 103 (*) and 60 (**) patients (respectively), because one patient underwent hormonal treatment prior to radical retropubic prostatectomy, so there was no postoperative Gleason score.

## Abbreviations

The following abbreviations are used in this manuscript:

ePLND	extended pelvic lymph node dissection
IQR	interquartile range
LN	lymph node
LNI	lymph node invasion
PCa	prostate cancer
PLND	pelvic lymph node dissection
PSA	prostate-specific antigen
SLN	sentinel lymph node
sLND	sentinel lymph node dissection
SPION	superparamagnetic iron oxide nanoparticles
SD	standard deviation
